# Metronidazole-induced encephalopathy in a patient with infectious colitis: a case report

**DOI:** 10.1186/1752-1947-5-63

**Published:** 2011-02-14

**Authors:** Hoon Kim, Young Woo Kim, Seoung Rim Kim, Ik Seong Park, Kwang Wook Jo

**Affiliations:** 1 Department of Neurosurgery, The Armed Forces Capital Hospital, Bundang, Korea; 2 Department of Neurosurgery, Bucheon St Mary's Hospital, College of Medicine, Catholic University, Bucheon, Korea

## Abstract

**Introduction:**

Encephalopathy is a rare disease caused by adverse effects of antibiotic drugs such as metronidazole. The incidence of metronidazole-induced encephalopathy is unknown, although several previous studies have addressed metronidazole neurotoxicity. Here, we report the case of a patient with reversible cerebellar dysfunction on magnetic resonance imaging, induced by prolonged administration of metronidazole for the treatment of infectious colitis.

**Case presentation:**

A 71-year-old Asian man, admitted to our hospital with hematochezia, underwent Hartmann's operation for the treatment of colorectal cancer three years ago. He was diagnosed with an infectious colitis by colonoscopy. After taking metronidazole, he showed drowsiness and slow response to verbal commands. Brain magnetic resonance imaging showed obvious bilateral symmetric hyperintensities within his dentate nucleus, tectal region of the cerebellum, and splenium of corpus callosum in T2-weighted images and fluid attenuated inversion recovery images. Our patient's clinical presentation and magnetic resonance images were thought to be most consistent with metronidazole toxicity. Therefore, we discontinued metronidazole, and his cerebellar syndrome resolved. Follow-up magnetic resonance imaging examinations showed complete resolution of previously noted signal changes.

**Conclusion:**

Metronidazole may produce neurologic side effects such as cerebellar syndrome, and encephalopathy in rare cases. We show that metronidazole-induced encephalopathy can be reversed after cessation of the drug. Consequently, careful consideration should be given to patients presenting with complaints of neurologic disorder after the initiation of metronidazole therapy.

## Introduction

Metronidazole is a commonly used antibiotic agent in various conditions such as anaerobic bacterial infections, protozoa infections (for example, giardiasis), *Helicobacter *associated gastritis, and hepatoencephalopathy. Previous reports have demonstrated that metronidazole toxicity may induce several neurologic side effects, including peripheral neuropathy, ataxic gait, dysarthria, convulsive seizures, and encephalopathy [1-4]. We describe the case of a patient with metronidazole-induced encephalopathy (MIE), with abnormalities found following brain magnetic resonance imaging (MRI), which had a succesful outcome after discontinuance of metronidazole.

## Case presentation

A 71-year-old Asian man, admitted with hematochezia, had previously been diagnosed with type 2 diabetes and underwent Hartmann's operation for the treatment of colorectal cancer three years ago. He was diagnosed with an infectious colitis by colonoscopy. After taking intravenous metronidazole for 14 days, he took oral metronidazole for 14 days, and was discharged home with oral metronidazole. Three days after discharge, he presented to our emergency room with drowsiness and slow response to verbal commands.

**Figure 1:**
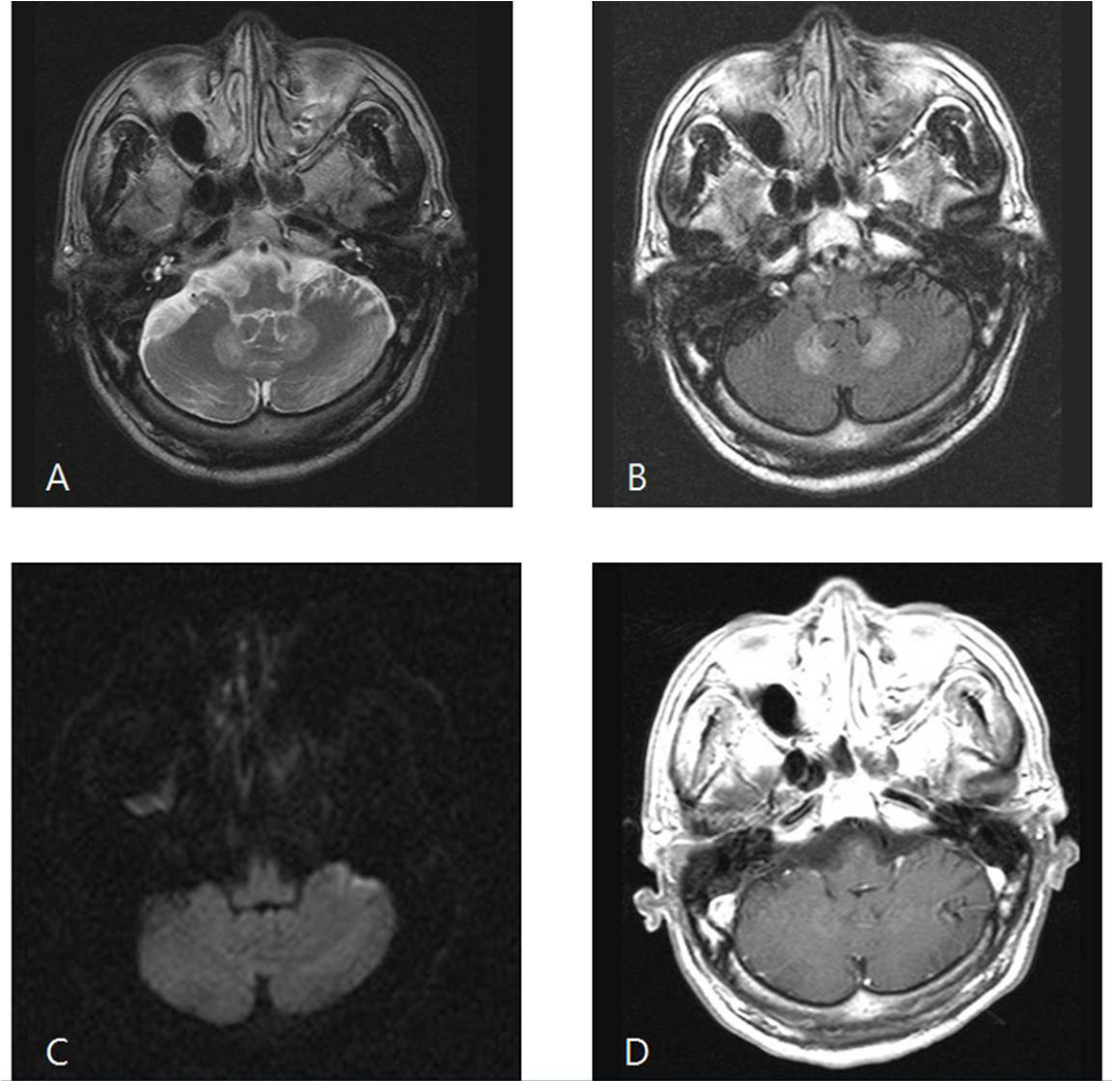
**Initial MRI findings**. A: T2-weighted image shows symmetrically increased signal intensities in the dentate nucleus of the cerebellum. B: FLAIR image shows symmetrically increased signal intensities in the dentate nucleus of the cerebellum. C: Diffusion weighted image shows no abnormality. D: Postgadolinium T1-weighted image shows no abnormality.

Neurological examination showed dysarthria, dysmetria on finger-to-nose examination, and an ataxic wide-based gait. Computed tomography (CT) performed on admission showed no evidence of acute hemorrhagic stroke and laboratory analysis was unremarkable. Thus, the preliminary diagnosis was cerebral infarction or metastatic disease. Our patient underwent brain magnetic resonance imaging (MRI). The results showed obvious bilateral symmetric hyperintensities within his dentate nucleus, tectal region of the cerebellum, and splenium of corpus callosum in T2-weighted images and fluid attenuated inversion recovery (FLAIR) images (Figure 1). The patient's clinical presentation and MRI images were thought to be most consistent with metronidazole toxicity. Therefore, we decided to discontinue metronidazole, and the patient's condition improved slowly.

**Figure 2:**
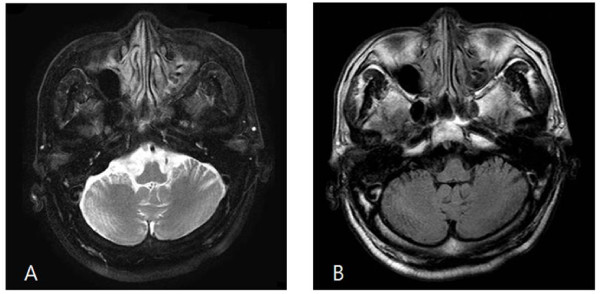
**A follow-up MRI three months after discontinuation of metronidazole shows complete resolution of the previously noted signal changes**. A: T2-weighted image. B: FLAIR image.

Three months after discontinuation of metronidazole, a follow-up examination showed that our patient's cerebellar syndrome had resolved. Follow-up MRI examination showed complete resolution of previously noted signal changes (Figure 2).

## Discussion

Metronidazole is available for treatment in anaerobic-related infections but may produce a number of neurologic side effects, such as cerebellar syndrome, encephalopathy, seizure, autonomic neuropathy, optic neuropathy, and peripheral neuropathy [2,3]. The incidence of MIE is unknown. The duration of treatment with metronidazole before cerebellar symptoms manifest is variable, and cumulative doses range from 25 g to 110 g [5]. In our case, total doses of metronidazole were 45.5 g.

The pathogenesis of metronidazole neurotoxicity is currently unknown and there are relatively few publications addressing the mechanism of metronidazole neurotoxicity. It has been suggested that metabolites of metronidazole may bind to RNA instead of DNA, possibly inhibiting RNA protein synthesis, which could potentially lead to axonal degeneration [6]. Another proposed mechanism involves the modulation of the inhibitory neurotransmitter gamma-aminobutyric acid (GABA) receptor within the cerebellar and vestibular systems [7].

Although the mechanism of metronidazole neurotoxicity remains unclear, most lesions induced by metronidazole neurotoxicity may be wholly reversible. The reversible changes associated with the acute toxic effects of metronidazole are most likely due to axonal swelling with increased water content rather than a demyelinating process. A further suggested mechanism involves vascular spasm that could produce mild reversible localized ischemia [4]. MRI in patients with MIE show that T2 hyperintense lesions in the cerebellar dentate nuclei are most commonly involved. The midbrain, dorsal pons, dorsal medulla, and corpus callosum can also be affected. Uncommon locations include the inferior olivary nucleus and the white matter of the cerebral hemispheres [4,8]. Lesions are always symmetric and bilateral, which is a typical pattern of metabolic encephalopathy. In each of the cases we reviewed, including ours, there was symmetrical increase of T2 signal intensity and absence of mass effect and enhancement. Reversible changes have previously been observed through MRI in the brains of patients with MIE [9].

In this case, our initial prediction - considering his underlying disease - was either cerebrovascular accident or metastatic cancer rather than drug-induced encephalopathy. However, his clinical history and MRI findings strongly suggested MIE. Our patient's symptoms resolved after cessation of the drug.

In the neurosurgical field, metronidazole is an alternative treatment for brain abscess in addition to surgical excision. Thus, a neurosurgeon should be able to recognize the adverse effects of metronidazole and a need for early diagnosis of MIE.

## Conclusions

Our case illustrates that metronidazole can cause reversible neurotoxicity. Appropriate neurological examinations, early diagnosis using MRI, and prompt cessation of the medication will lead to a better prognosis. Therefore, awareness of the potential neurological side effects of metronidazole and an accurate clinical impression of the attending physician is very important in metronidazole-induced encephalopathy.

## Consent

Written informed consent was obtained from the patient for publication of this case report and any accompanying images. A copy of the written consent is available for review by the Editor-in-Chief of this journal.

## Competing interests

The authors declare that they have no competing interests.

## Authors' contributions

HK provided the case information, and was a major contributor to the case and discussion section of the paper. YWK, SRK and ISP interviewed the patient, reviewed the medical records and wrote the case presentation. KWJ provided major contributions to the case presentation and discussion sections, and edited the final manuscript. All authors read and approved the final manuscript.
